# Case Report of a Pregnancy During Ipilimumab Therapy

**DOI:** 10.1200/JGO.17.00019

**Published:** 2017-08-22

**Authors:** Andrew Mehta, Kevin B. Kim, David R. Minor

**Affiliations:** All authors: California Pacific Medical Center, San Francisco, CA.

## INTRODUCTION

The most common malignancy during pregnancy is melanoma, and approximately one third of all women diagnosed with melanoma are of childbearing age.^1,2^ Thus, the effects of the drugs used in the treatment of melanoma on reproduction and development is of high interest. Ipilimumab, an anti–cytotoxic T-cell lymphocyte-4 (CTLA-4) antibody, was approved for the therapy of metastatic melanoma in 2011. We describe what we believe is the first report with follow-up of the successful outcome of a pregnancy in a patient who received ipilimumab therapy for metastatic melanoma.

## CASE REPORT

A 31-year-old female was found to have a 5.8-mm-deep melanoma on biopsy of a right-side calf lesion in May 2011. She had a wide local excision and sentinel lymph node biopsy 1 month later that showed two positive inguinal lymph nodes. A positron emission tomography scan showed no evidence of distant disease; the patient declined both completion node dissection and adjuvant therapy. In July 2012, she developed multiple in-transit metastases that involved the cutaneous and subcutaneous tissues in the right-side thigh. Her tumor was found to harbor a *BRAF* V600E mutation, and she started vemurafenib treatment. The patient had a partial response, but by June 2013, the disease progressed with three new lesions on the lateral right-side thigh and knee. No distant metastases were present on imaging. She began treatment with ipilimumab 3 mg/kg every 3 weeks on June 28, 2013, with her third dose delayed until September 9 as a result of grade 1 diarrhea. Because of an increase in the number of in-transit lesions by August 30, the patient also received biweekly intralesional aldesleukin (interleukin 2) injections of 9 million International Units total dose each on five occasions during September. Aldesleukin was added in the hope of synergistic efficacy and caused no significant adverse effects.

Although the patient was told to avoid pregnancy, she discovered she was pregnant after her third dose of ipilimumab. Ultrasound confirmed a pregnancy of 6 weeks gestation. She was counseled about potential clinical benefits and risks of congenital malformations to the fetus from ipilimumab and interleukin 2; she declined termination for religious reasons. The patient and her physician decided to proceed with a fourth dose of ipilimumab in October; the patient then received eight additional doses of low-dose intralesional aldesleukin, which finished in November. At that point, her physician believed the risks to the fetus of additional aldesleukin outweighed the possible benefits. The patient had negative chest x-ray results on February 7, 2014; otherwise, the status of her in-transit disease was assessed clinically without imaging during her pregnancy because the disease was visible or palpable. By February 14, progressive disease was evident and she had five in-transit metastases (5 to 12 mm in size) surgically removed. Thyroid test results during and after immunotherapy were normal. The patient had no autoimmune adverse effects from ipilimumab other than grade 1 diarrhea.

She delivered a healthy male infant on May 21, 2014. No special assessment of the placenta was done for metastatic disease. Routine follow-up of her child at 2 3/4 years of age showed a healthy boy with no physical or developmental abnormalities and no history of unusual infections. Detailed testing of the child’s immune system has not been performed.

After delivery, the patient was found to have new large metastases to her right-side inguinal and iliac lymph nodes and a recurrent in-transit metastasis. In June 2014 she was started on pembrolizumab, but unfortunately had further progression of disease with distant metastases in 2016.

## DISCUSSION

Ipilimumab is a human monoclonal anti–CTLA-4 antibody (immunoglobulin G1) that blocks the inhibition of T-cell activation by the CTLA-4 protein on the surface of T lymphocytes. This results in proliferation and activation of effector T cells, inhibition of regulatory T cells, and expansion of the T-cell repertoire. Ipilimumab was approved by the US Food and Drug Administration for metastatic melanoma in 2011 and approved for adjuvant therapy for stage III melanoma in 2016.^3^

In animal studies, cynomolgus monkeys were given 2.6 to 7.2 times the recommended dose of 3 mg/kg ipilimumab and were found to have an increased incidence of third trimester miscarriage, stillbirth, premature delivery, low birth weight, and infant mortality. No adverse effects were detected in the first two trimesters. One female infant monkey developed unilateral renal agenesis of the left-side kidney and ureter, and one male infant had an imperforate urethra. Ipilimumab is pregnancy category C.^4^

No adequate studies of ipilimumab have been conducted in human pregnancy. The US Food and Drug Administration Adverse Event Reporting System database contains seven cases of maternal exposure to ipilimumab from January 2008 through June 2016 (personal communication, D Minor, January 2017). Reported outcomes include one spontaneous abortion in a patient who also received dabrafenib, one stillbirth, one ectopic pregnancy, two pregnancies terminated by induced abortions, and two pregnancies with unknown outcomes. At this time, the database does not contain reports of birth defects or congenital anomalies.

Checkpoint blockers that affect the PD-1 pathway may have a higher risk of fetal loss or birth defects than ipilimumab as a result of the role of that pathway in protecting the placenta and fetus from attack by the mother’s immune system.^5^ Given that pregnancy induces a relative immunosuppressed state that protects the fetus, melanoma arguably can progress more rapidly during pregnancy, although no conclusive evidence proves this theory. No known mechanism by which pregnancy directly influences melanoma growth exists, and the prognosis in the case of melanoma does not appear to be affected by pregnancy.^6^ The current patient’s melanoma progressed while receiving treatment during pregnancy; however, what role her pregnancy played in this progression is unknown.

This case report shows the healthy outcome of a child exposed in utero to ipilimumab and low-dose intralesional aldesleukin administered for metastatic melanoma. It and others may contribute to our knowledge of how to advise patients who develop an unplanned pregnancy while receiving ipilimumab.
